# Network Pharmacology Combined with Experimental Validation to Investigate the Mechanism of the Anti-Hyperuricemia Action of *Portulaca oleracea* Extract

**DOI:** 10.3390/nu16203549

**Published:** 2024-10-19

**Authors:** Yiming Zhang, Shengying Zhu, Yueming Gu, Yanjing Feng, Bo Gao

**Affiliations:** 1 School of Life Sciences, Jilin University, Changchun 130012, China; yimingz21@mails.jlu.edu.cn (Y.Z.); zhusy22@mails.jlu.edu.cn (S.Z.); guym22@mails.jlu.edu.cn (Y.G.); fengyj23@mails.jlu.edu.cn (Y.F.); 2 Key Laboratory for Molecular Enzymology and Engineering, Jilin University, Ministry of Education, Changchun 130012, China

**Keywords:** hyperuricemia, *Portulaca oleracea*, xanthine oxidase, ABCG2, berberine

## Abstract

Background/Objectives: Hyperuricemia (HUA) is a common metabolic disease caused by purine metabolic disorders in the body. *Portulaca oleracea* L. (PO) is an edible wild vegetable. Methods: In this study, the regulatory effect of PO on HUA and its potential mechanism were initially elucidated through network pharmacology and experimental validation. Results: The results showed that PO from Sichuan province was superior to the plant collected from other habitats in inhibiting xanthine oxidase (XOD) activity. Berberine and stachydrine were isolated and identified from PO for the first time by UPLC-Q-Exactive Orbitrap MS. The potential molecular targets and related signaling pathways were predicted by network pharmacology and molecular docking techniques. Molecular docking showed that berberine had strong docking activity with XOD, and the results of in vitro experiments verified this prediction. Through experimental analysis of HUA mice, we found that PO can reduce the production of uric acid (UA) in the organism by inhibiting XOD activity. On the other hand, PO can reduce the body ‘s reabsorption of urate and aid in its excretion out of the body by inhibiting the urate transporter proteins (GLUT9, URAT1) and promoting the high expression of urate excretory protein (ABCG2). The results of H/E staining showed that, compared with the positive drug (allopurinol and benzbromarone) group, there was no obvious renal injury in the middle- and high-dose groups of PO extract. Conclusions: In summary, our findings reveal the potential of wild plant PO as a functional food for the treatment of hyperuricemia.

## 1. Introduction

With the improvement of modern living standards, people‘s dietary patterns have begun to develop towards high purines, and the number of patients with hyperuricemia (HUA) has, accordingly, also increased [1,2]. Many studies have shown that HUA is due to the disorder of the purine metabolism or the obstruction of UA excretion in the body, which eventually leads to a high concentration of UA in serum, which then evolves into a metabolic disease that endangers human health [3,4].

Uric acid (UA) is the end-product of a purine metabolism series in the body [5]. UA homeostasis is maintained by multiple organs in the body. After UA is formed, about 1/3 of it is excreted through the gastrointestinal tract and 2/3 is excreted through the kidney tissue [6]. Eventually, the remainder is reabsorbed into the bloodstream [7]. Researchers have found that with an increase of UA levels, urate crystals gradually formed in the tubules and interstitia of the kidneys, ultimately leading to pathological damage to kidney tissue [8,9]. Excessive accumulation of urate may trigger diseases such as gout, which seriously affects the daily lives of patients [10].

The main factor contributing to the cause of HUA is a dysfunction of urate transport in the kidneys or a blockage of the transport pathway. In renal tissues, urate transporter proteins include URAT1 (SLC22A12), OAT4 (SLC22A11), OAT10 (SLC22A13), and GLUT9 (SLC2A9). Excretory proteins include ABCG2, ABCC4, and NPT1 (SLC17A1). OAT1 (SLC22A6), OAT2 (SLC22A7), and OAT3 (SLC22A8) proteins, on the outside of the cellular basement membrane, are similarly involved in urate transport [11,12,13,14,15]. In addition, XOD, as a key enzyme in UA production, is often considered an important target in HUA studies [16]. Allopurinol, benzbromarone, and febuxostat, which are widely used in clinical practice, have been associated with serious adverse effects [17,18,19,20]. In contrast, natural drug preparations often have the advantages of multi-target regulation and low levels of side-effects, and have shown great potential in the field of HUA treatment in recent years [21,22,23].

Portulaca oleracea L. (PO) is an annual herb of Caryophyllaceae and Portulacaceae, with fleshy, branched, and light-red stems. It is distributed all over the world, mainly in temperate and tropical regions, and is distributed throughout many provinces in China. It is an edible wild plant [24]. People in many countries, such as Spain, Greece, Italy, Turkey, the United States, and China, cook it as a traditional dish. It is one of the famous medicinal and edible wild plants in China, one which is called the “longevity vegetable” in traditional folklore [25,26,27,28]. PO is rich in a variety of bioactive substances, including polysaccharides, alkaloids, unsaturated fatty acids, flavonoids, terpenoids, proteins, vitamins, and minerals [29,30]. In ancient Chinese traditional prescriptions, it has the functions of detoxification, detumescence, anti-inflammation, diuresis, and so on. In folk medicine, it is used to treat bloody diarrhea [28,31]. In recent years, studies have found that the extracts of PO have a wide range of pharmacological activities, including anti-inflammatory [32], anti-diabetic [33], anti-bacterial [34], anti-ulcer [35], anti-oxidation, and immunomodulatory effects [36]. However, there have not yet been any clinical reports demonstrating its efficacy in lowering UA.

Within the realm of traditional Chinese medicine, considerable emphasis is placed on authentic medicinal materials, denoting those cultivated in specific regions and possessing superior therapeutic efficacy and a more stable quality compared with the same herbs from other origins. Consequently, we meticulously chose four emblematic regions across China, varying in both longitude and latitude, specifically, Sichuan, Henan, Guangdong, and Jilin, to meticulously analyze and juxtapose the inhibitory prowess of PO against XOD, whilst theoretically forecasting PO’s plausible targets in HUA treatment through the application of LC-MS technology, network pharmacology, and molecular docking. Finally, the pertinent mechanism was further elucidated by in vivo experiments.

## 2. Materials and Methods

### 2.1. Materials

The aerial parts of PO were harvested from four distinct locations: Mianyang City, Sichuan Province (104°44′ E, 31°53′ N); Luoyang City, Henan Province (112°4′ E, 34°20′ N); Guangzhou City, Guangdong Province (113°15′ E, 23°06′ N); and Changchun City, Jilin Province (125°42′ E, 31°53′ N). These specimens were meticulously identified by Professor Shuwen Guan of the College of Life Sciences, Jilin University.

XOD and xanthine (XA) were purchased from Shanghai Yuanye Bio-Technology Co., Ltd. (Shanghai, China). Methanol and formic acid were purchased from ThermoFisher, Waltham, MA, USA. Berberine (HPLC ≥ 98%) and stachydrine (HPLC ≥ 98%) were purchased from Aladdin Reagent Co., Ltd. (Shanghai, China). Yeast extract was purchased from Beijing Oberstar Biotechnology Co., Ltd. (Beijing, China). Allopurinol was purchased from Jiangsu World Trade Tianjie Pharmaceutical Co., Ltd. (Yancheng, China). Benzbromarone was purchased from Herman Pharma Kft. (Hungary, Germany). UA, XOD, blood urea nitrogen (BUN), and serum creatinine (SCr) kits were purchased from Nanjing Jiancheng Bioengineering Institute (Nanjing, China). The following primary antibodies were purchased from Proteintech Group, Inc. (Wuhan, China): ABCG2 (27286-1-AP, 1:1000), GLUT9 (26486-1-AP, 1:600), and URAT1 (14937-1-AP, 1:1500). The β-actin (GB15003, 1:2000) and horseradish peroxidase (HRP)-labeled anti-rabbit IgG (GB23303, 1:5000) were purchased from Wuhan Servicebio Technology Co., Ltd. (Wuhan, China).

### 2.2. Preparation of PO Extract

The aerial parts of freshly picked PO were dried and crushed, and then passed through a 60-mesh sieve. PO powder was mixed with distilled water at a ratio of 1:12 (*w*/*v*) and extracted at 60 °C for 3 h. During the extraction, PO water extract and water-extract residue were obtained by ultrasonic-assisted extraction and vacuum filtration. Then, ethanol was used to extract the water extraction-filter residue under the same conditions to obtain the ethanol extract. The water extract and the ethanol extract were then mixed and, following this, concentrated using a vacuum rotary evaporator, and the PO extract was obtained after vacuum drying. Subsequently, the extract was resuspended in ultrapure water for subsequent experiments.

### 2.3. XOD Inhibition-Ability Experiment

Based on the formation of UA catalyzed by XOD, a stable enzymatic reaction system was established [37]. Firstly, 0.08 U/mL XOD was added to the system containing substrate (1.5 mm xanthine, XA) and inhibitor (sample concentrations were 1000, 500, 400, 250, 200, and 100 μg/mL, respectively), and then the catalytic reaction was carried out at 37 °C for 30 min. Subsequently, the absorbance was measured by a spectrophotometer at 295 nm, and the XOD inhibition rate was calculated according to the following formula:

$$XOD\;Inhibition\;rate\;\left(\%\right)=\frac{\left(A-B\right)-\left(C-D\right)}{A-B}\times 100\%$$

A: OD295 nm solution containing XA and XOD.

B: OD295 nm solution containing only XA.

C: OD295 nm solution containing inhibitors, XA, and XOD.

D: OD295 nm solution containing inhibitors and XA.

### 2.4. Compositional Analysis

PO extracts were identified by UPLC-Q-Exactive Orbitrap MS [38]. A sample of 0.15 g was weighed, and 1000 μL 80% methanol and grinding beads were added. Grinding proceeded for 5 min, and then the mixture was vortexed for 10 min. During the centrifugation at 4 °C for 10 min, the centrifugal force was 20,000× *g*. The supernatant was then filtered and injected for analysis.

An Ultimate AQ-C18 chromatographic column (150 × 2.1 mm, 1.8 μm, Welch Technology (SHANGHAI) Co., Ltd., Shanghai, China) was used in this study. The mobile phase A was composed of 0.1% (*v*/*v*) formic acid and water. The B phase was methanol. Gradient elution was performed under the following conditions: 98% A phase (0–1 min), 98–80% A phase (1–5 min), 80–50% A phase (5–10 min), 50–20% A phase (10–15 min), 20–5% A phase (15–20 min), 5% A phase (20–27 min), 5–98% A phase (27–28 min), and 98% A phase (28–30 min). The fixed flow rate was 0.30 mL/min, the temperature of the automatic sampler was 10.0 °C, the column temperature was 35 °C, and the injection volume was 5.00 μL. The mass spectrometer was equipped with a Q Exactive ESI source with a scan range of *m*/*z* 150–2000. The detection method selected was data-dependent tandem mass spectrometry (dd-MS2) for full-mass scanning. The resolution of the full-mass scanning was 70,000, and the resolution for the dd-MS2 was 17,500. The voltage of the ion jet needle was 3.2 kV (positive). The capillary temperature was 300 °C. The collision gas was high-purity argon (purity ≥ 99.999%). The sheath gas was nitrogen (purity ≥ 99.999%) 40 Arb; the auxiliary gas was nitrogen (purity ≥ 99.999%), 15 Arb; and the heater temperature was 350 °C. The data acquisition time was 30.0 min.

The analysis of berberine and stachydrine in the PO was based on the methods of Ren et al. [39] and Yan et al. [40]. Berberine (HPLC ≥ 98%) and stachydrine (HPLC ≥ 98%) were dissolved in methanol and treated with ultrasound for 5 min to prepare a standard stock solution, with a concentration of 5 μg/mL, for subsequent analysis.

### 2.5. Identification of Candidate Targets of PO with Effects on HUA

The PO potential targets were predicted by the TCMSP database [41] (https://tcmsp-e.com/index.php/, accessed on 12 March 2024), Targetnet database [42], and SwissTargetPrediction [43] (http://www.swisstargetprediction.ch/, accessed on 12 March 2024). The above collected targets were merged and de-weighted using the UniProt database [44] (http://www.uniprot.org, accessed on 12 March 2024) and further standardized into official gene names for subsequent analysis. Employing “hyperuricemia” as the search term, disease-associated targets were explored within the CTD [45] (http://ctdbase.org/, accessed on 12 March 2024), OMIM [46] (https://omim.org/, accessed on 12 March 2024), and DisGeNET databases [47] (https://www.disgenet.org/, accessed on 12 March 2024), with the results subsequently being merged and de-duplicated to procure hyperuricemia-associated targets. Finally, the Venny2.1.0 online utility (https://bioinfogp.cnb.csic.es/tools/venny, accessed on 12 March 2024) was employed for visual examination of the intersecting elements between the disease-associated and PO targets. The members of the intersecting target ensemble were regarded as putative therapeutic targets for both PO and HUA.

### 2.6. Analysis of Protein−Protein Interaction Network

The collected intersecting targets were imported into the String database [48] (https://cn.string-db.org/, accessed on 12 March 2024), the free genes were removed, and confidence levels were selected to obtain a PPI network graph for PO treatment of HUA, in which the nodes represent the potential targets and the connecting lines represent their interactions. The nodes were saved and imported into Cytoscape 3.7.1 software [49] for visualization and analysis, and the importance of the nodes in the network was evaluated by using the degree value after the topological network analysis; the larger the value of the degree was, the greater was the relevance and the more effective the effect of the node in the network.

### 2.7. Construction of Gene Enrichment Analysis

The bioinformatics analysis platform DAVID database [50] (https://david.ncifcrf.gov/, accessed on 13 March 2024) was used to perform GO annotation of biological processes, cellular components, and molecular functions, in addition to KEGG pathway enrichment analysis of the above PO and HUA intersection targets. The obtained results were arranged in descending order according to their −lg(P) values, and the results were visualized and analyzed using the microbiology platform (https://www.bioinformatics.com.cn/login/, accessed on 13 March 2024) in order to explore the relevant mechanism of action of PO in treating HUA.

### 2.8. Molecular Docking Validation

The 3D structural information associated with the berberine molecule was downloaded using the PubChem database [51] (pubchem.ncbi.nlm.nih.gov/, accessed on 13 March 2024), and the XOD protein and its ligand complexes were obtained from the PDB protein structure database (htps://www.rcsb.org/, accessed on 13 March 2024); water molecules and ligands of target proteins were removed using PyMOL 2.5.7 software, the 3D structure of SDF was converted to PDB format, and the receptor and ligand were pre-processed using AutbDock Tools and then saved in PDBQT format for use. Finally, molecular docking of the ligand and receptor was performed using AutoDock 4.2.6 software [52]. It is generally accepted that when the docking score is less than −7.0, it indicates significant binding activity between the active ingredient and the target; less than −5.0 indicates good binding activity between the two; less than −4.25 indicates the presence of some binding activity [53].

### 2.9. Establishment of HUA Mouse Model

All experimental procedures involving animals adhered strictly to the guiding principles for animal care and use outlined by the Animal Experiment Ethics Committee of Jilin University (Changchun, China), in accordance with the regulations stipulated by the Committee for Animal Use and Care and the principles established in the Helsinki Declaration. SPF-grade male C57BL/6J mice, aged 4–6 weeks, weighing 20 ± 2 g, were purchased from Liaoning Changsheng Biotechnology Co., Ltd. (Shenyang, China), under license number SCXK (Liao) 2020-0001. The experimental animals were not genetically modified during the experiment. The mice were accommodated in the Jilin University Experimental Animal Platform, where they had unrestricted access to sterile water and food. The animals were maintained under controlled conditions of (22 ± 2) °C and 60% humidity, and subjected to a 12 h light/dark cycle. After 1 week of adaptive feeding, the mice were randomly divided into groups. The change in body weight of each mouse was recorded for subsequent equivalent dose conversion based on body weight.

Referring to the method of Dai et al. [54], the HUA mouse model was induced using gastric gavage with yeast paste. Except for the control group (*n* = 8), the mice received daily gastric gavage of yeast paste at a dosage of 20 g/kg. After 14 days of pretreatment, the mice were randomly divided into six groups as follows: the model group; the allopurinol group (7.6 mg/kg); the benzbromarone group (7.6 mg/kg); and low-, medium-, and high-dose PO groups (0.5, 1, and 2 g/kg, LPO, MPO, HPO, respectively) (n = 8 per group). Following this, daily gastric gavage with yeast paste was performed every morning to sustain clinical manifestations of HUA, followed by daily drug administration for 7 consecutive days in the afternoon. On the final day of treatment, mice were euthanized with carbon dioxide 2 h after administration. Blood samples were collected and centrifuged at 3000 rpm, 4 °C for 15 min to separate serum. The levels of UA, XOD, creatinine (SCr), and urea nitrogen (BUN) in serum were detected by detection kit. Following extraction, mice liver and kidney tissues were rinsed with physiological saline, air-dried, and weighed post-absorption of moisture; the left kidney tissues were fixed in 4% paraformaldehyde for histopathological examination, and the right kidney tissues were stored at −80 °C for subsequent analysis.

### 2.10. Western Blot Analysis

Kidney tissues were subjected to lysis utilizing RIPA buffer supplemented with protease inhibitors. Protein concentration was quantified employing the bicinchoninic acid (BCA) protein assay kit. Subsequently, proteins were fractionated via sodium dodecyl sulfate-polyacrylamide gel electrophoresis (SDS-PAGE) and subsequently transferred onto polyvinylidene difluoride (PVDF) membranes. Following blocking with 5% non-fat milk for 2 h, the membranes were then incubated overnight at 4 °C with antibodies targeting ABCG2, GLUT9, and URAT1. Subsequent to this, the membranes were exposed to secondary antibodies for a duration of 1.5 h. Lastly, the membranes underwent washing and imaging utilizing an enhanced chemiluminescence (ECL) detection kit. Band intensities were quantified utilizing Image J software (version 1.54).

### 2.11. H/E Staining and IHC Analysis

The kidney tissues, fixed and embedded, underwent precision slicing into 4 μm sections. Following meticulous deparaffinization employing a gradient of xylene and ethanol, the sections underwent staining with hematoxylin/eosin (H/E).

Upon deparaffinization, endogenous peroxidase activity was effectively inhibited using 3% hydrogen peroxide, and subsequent blocking of the sections was achieved with 3% BSA. Subsequently, the sections were incubated overnight at 4 °C with primary antibodies targeting ABCG2, GLUT9, and URAT1. Following this, the sections were subjected to a 1 h incubation at room temperature with secondary antibodies, followed by detection utilizing DAB chromogen and subsequent counterstaining with hematoxylin.

### 2.12. Statistical Analysis

The values are expressed as the mean ± SEM/SD in multigroup animal experiments. One-way ANOVA was used to analyze the significance of differences, and a value of *p* < 0.05 was considered statistically significant; highly significant differences were indicated at *p* < 0.01. All statistical analyses were performed using GraphPad Prism 8 software. IHC and Western Blot analyses were performed by Image J, and subsequently employed for densitometric analysis.

## 3. Results

### 3.1. In Vitro XOD Inhibition Experiment

**Figure 1:**
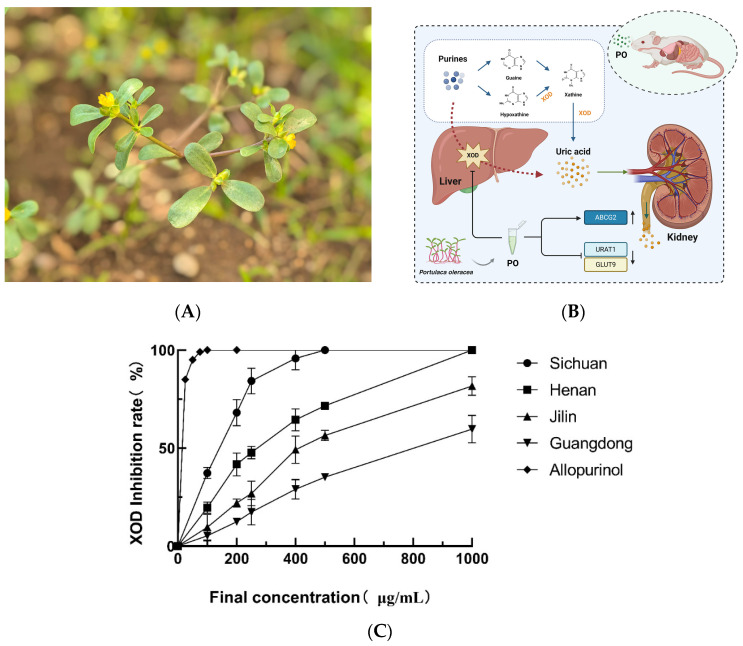
(**A**) Photograph of the aerial parts of *Portulaca Oleracea*. (**B**) Mechanism map of purine metabolism and uric acid excretion pathway (Created in bioRender). (**C**) Inhibitory effect of PO extracts from different producing areas on XOD, in vitro.

Figure 1 demonstrates the inhibition effect of PO on XOD for the Sichuan, Henan, Guangdong, and Jilin samples; the degree of inhibition of XOD by PO extracts from different geographical regions and different concentrations was calculated based on the absorbance (OD) at 295 nm of the reaction systems of each group. The corresponding IC50 inhibition curves were plotted using GraphPad Prism 8. According to the enzyme inhibition rate curves, it can be seen that the PO extracts showed concentration dependence, and the best inhibition of XOD was achieved by the PO from Sichuan, with an IC50 value of 160 μg/mL; we therefore chose the PO from Sichuan for the subsequent experiments.

### 3.2. Analysis of PO Components from Sichuan

**Figure 2:**
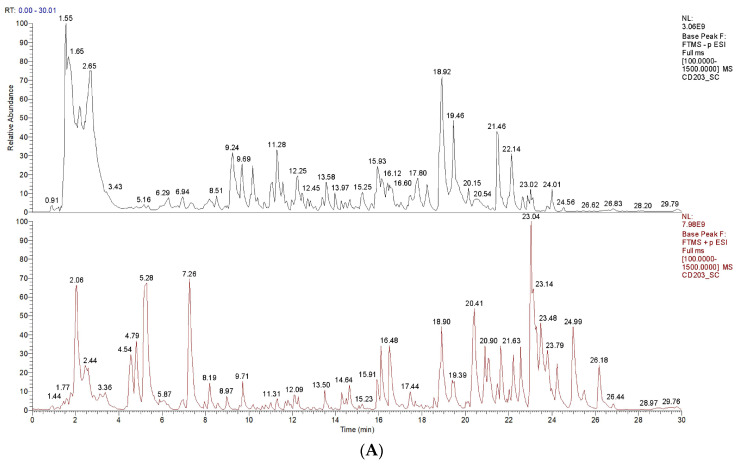

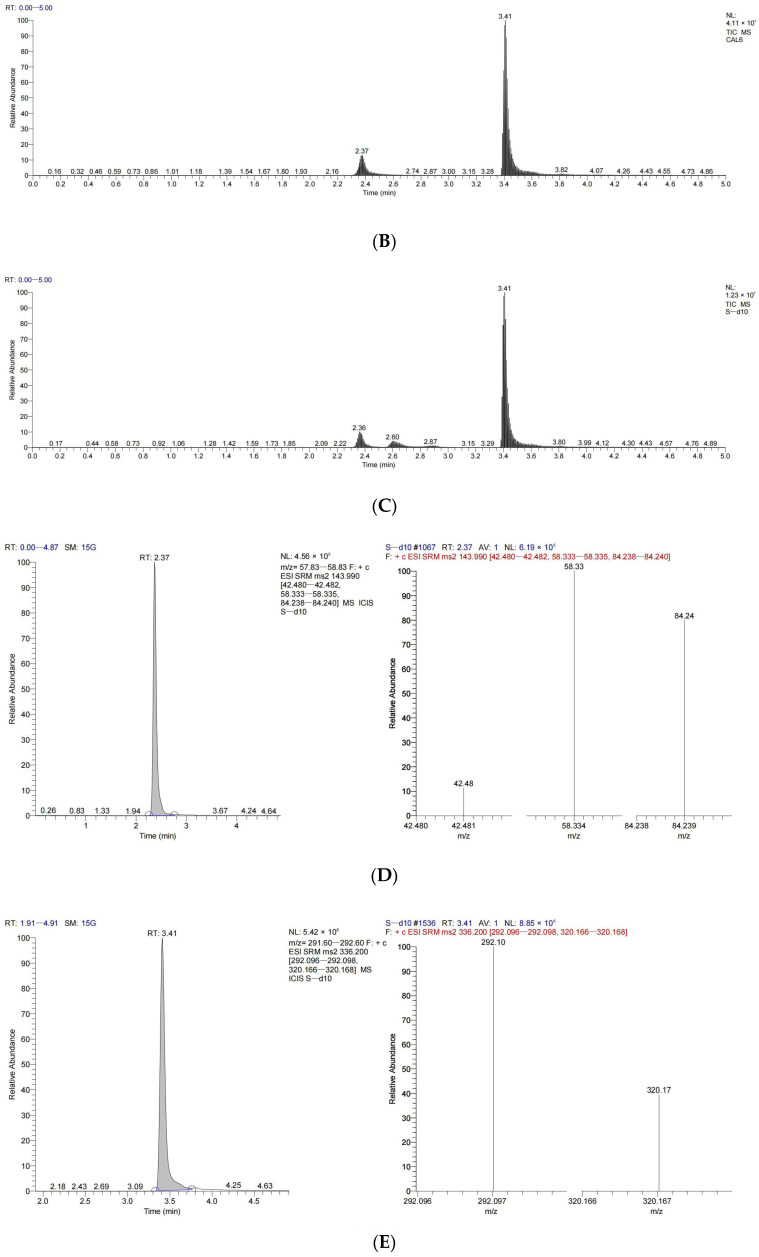
PO component analysis by UPLC-Q-Orbitrap MS. (**A**) Total ion chromatogram (TIC). (**B**) LC-MS chromatograms of the stachydrine (RT: 2.37) and berberine (RT: 3.41) standards. (**C**) LC-MS chromatograms of the stachydrine (RT: 2.36) and berberine (RT: 3.41) in the sample. (**D**) LC-MS chromatograms of the stachydrine ion channel (**left**) and MS2 spectra (**right**) for the separated sample. (**E**) Ion channel chromatogram (**left**) and MS2 spectra (**right**) of berberine in the separated sample.

UPLC-Q-Orbitrap MS was used to characterize the major components in the PO extract in both positive and negative ion modes. The total ion chromatogram of PO is shown in Figure 2A. The compounds with the best matching score for mzCloud (above 90) in the possible molecular formula deduced from the high-resolution mass spectrometry information were selected and analyzed, in combination with data from the literature. Trigonelline, betaine, stachydrine, berberine, salsolinol, baicalin, tangeritin, nobiletin, curcumin, scopoletin, vanillin, lupeol, cafestol, oleanolic acid, salsolinol, and other components were screened. Among them, stachydrine and berberine were, for the first time, detected in PO, in the sample produced in Sichuan. Berberine has broad-spectrum antibacterial activity and can potentially be used as a drug for the treatment of various diseases [55]. Stachydrine is a multifunctional bioactive substance with great potential in the treatment of many diseases [56].

In order to verify the presence of stachydrine and berberine in PO samples more accurately, they were compared with the corresponding standards using the LC-MS technique. In Figure 2B,C, the retention times of the samples showed RT1 = 2.36 min and RT2 = 3.41 min, values which were consistent with the retention times of the standards, which were Rt = 2.37 and 3.41 min, respectively. Furthermore, in the MS2 spectra shown in Figure 2D,E, target compound 1 was found to produce ions at *m*/*z* 144.10 [M + H], *m*/*z* 84.24, *m*/*z* 58.33 and *m*/*z* 42.48, yielding fragment ions in agreement with the stachydrine standard comparison. Similarly, target compound 2 produced fragment ions at *m*/*z* 336.12 [M + H], *m*/*z* 320.17, and *m*/*z* 292.10, in agreement with the berberine standards. Eventually, the two compounds were identified as stachydrine and berberine.

### 3.3. Construction of the Component–Target–Disease Network and Analysis of the PPI Network

**Figure 3:**
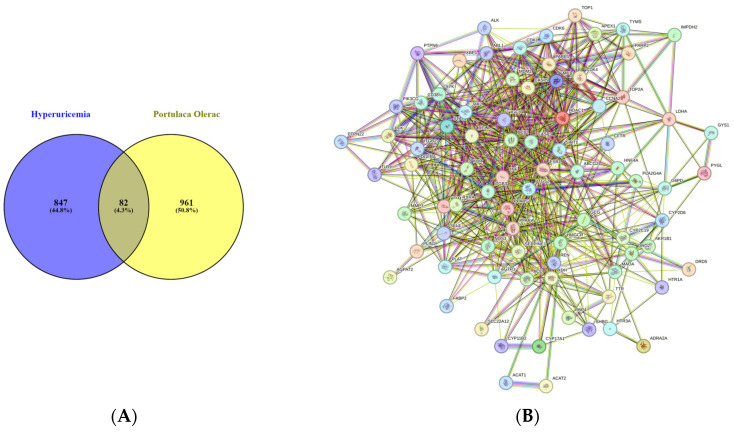

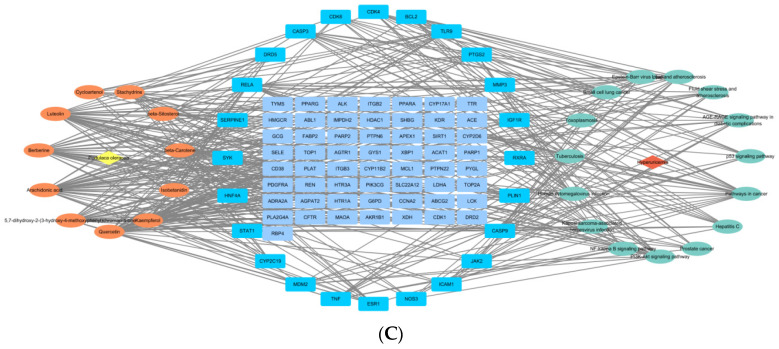
PPI network of PO active-compound targets. (**A**) Venn diagram of active compounds in PO and intersection targets in HUA. (**B**) Potential-target PPI network diagram. (**C**) Component–target–pathway network diagram for PO in the treatment of HUA.

A total of 82 cross-targets were screened by the Venn intersection of PO component-related targets and HUA disease-related targets which had been identified as potential therapeutic targets for HUA (Figure 3A). The interactions between target proteins are shown in Figure 3B. The PPI network contained 82 nodes and 712 edges. After processing, a total of 15 key targets were obtained: ABCG2, PPARG, HMGCR, CASP3, PARP1, MCL1, BCL2, ESR1, TNF, ACE, SIRT1, ICAM1, REN, PTGS2, GCG. Through the analysis of these key targets, it was found that the XDH (XOD), ABCG2 protein and its associated SLC22A12 (URAT1) protein were closely related to urate transport, anion transport, small molecule transport, and purine-containing compound metabolism, which are common targets in HUA research. Therefore, we selected proteins such as ABCG2 and URAT1 for further study.

The intricate network depicting the components, targets, and pathways influenced by PO in addressing HUA is visually represented in Figure 3C. The left side, depicted in orange, signifies the active constituents present in PO, while the right side, in green, delineates the pertinent signaling pathways identified through KEGG enrichment analysis. The intersecting targets of these components and pathways are depicted in blue. Subsequent topological analysis, facilitated by plugins, unveiled potential biological impacts of PO on HUA, targeting key elements like RELA, CASP9, BCL2, CASP3, CDK4, and TNF. These interactions are implicated in pivotal signaling cascades, encompassing the cancer, PI3K-Akt, p53, and NF-κB pathways, among others.

### 3.4. Enrichment Analysis of Related Pathways and the Biological Process

**Figure 4:**
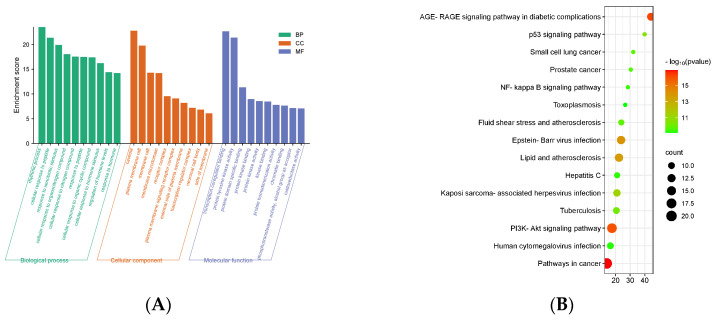

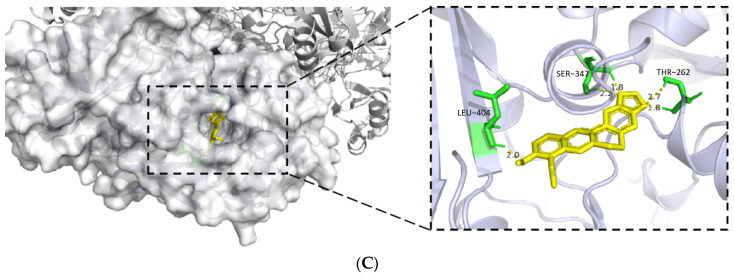
(**A**) GO function annotation. (**B**) KEGG pathway enrichment analysis. (**C**) Visualization of berberine and XOD molecular docking results. The white structure represents the ABCG2 protein, the yellow structure represents the active compound, and the green structure represents the binding site between the two. The value represents the binding affinity, and the unit is kcal/mol.

The comprehensive GO analysis resulted in the identification of a remarkable 1124 entries related to biological processes (BP), encompassing a diverse array of phenomena ranging from rhythmic processes to intricate hormonal regulatory mechanisms and responsive reactions to organic substances, among others (as illustrated in Figure 4A). Moreover, the analysis unveiled 46 distinct entries associated with cellular components (CC), spanning crucial entities like the cytoplasm, receptors, and extracellular regions. Additionally, it elucidated 97 entries pertaining to molecular functions (MF), exemplified by crucial interactions such as protein kinase binding, chromatin binding, and oxidoreductase activity, among others.

Subsequent enrichment analysis of KEGG pathways unveiled their intricate association with various critical signaling cascades, notably encompassing cancer-related pathways, the AGE-RAGE signaling pathway implicated in diabetic complications, the pivotal PI3K-Akt signaling cascade, the regulatory p53 signaling pathway, and the intricate NF-κB signaling pathway, among others (as depicted in Figure 4B).

### 3.5. Molecular Docking and Residue Interaction

With the aid of PyMOL software, the binding pocket of the berberine–XOD complex was visualized (Figure 4C), having a binding energy of approximately −10.00 kJ-mol^−1^, in which amino acid residues located within approximately 4.0 Å (Å) of the berberine have been highlighted. After careful screening, we have targeted a series of key active site residues, including THR262, SER347, and LEU404, which are uniquely located and surrounded near the active center of molybdenum chalcogenide, and the results indicate that berberine has strong docking activity with XOD. The binding energy of stachydrine with XOD was about −3.51 kJ-mol^−1^, which is a weak binding ability. Therefore, according to the experimental steps described in Section 2.3, the IC50 value of berberine was about 74 μg/mL, demonstrating a good inhibitory effect on XOD in vitro (Figure S1).

### 3.6. In Vivo Studies on the Reduction of Uric Acid

**Figure 5:**
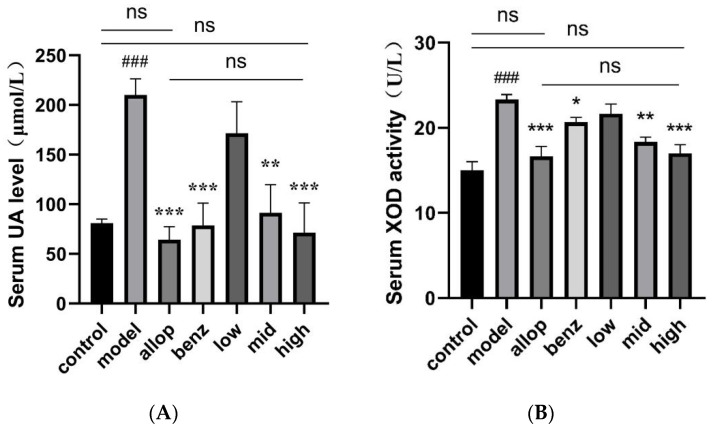
Serum levels of UA and XOD in mice after intragastric administration. (**A**) Serum uric acid level in mice after intragastric administration. (**B**) The level of XOD in serum of mice after intragastric administration. ^###^ *p* < 0.001 compared with the control group. * *p* < 0.05, ** *p* < 0.01, and *** *p* < 0.001, compared with the model group. ns (no significance).

UA levels in serum samples from each group of mice were measured using the colorimetric method to compare the changes in serum-UA levels in mice after drug administration. After 14 days of modeling, compared with the control group, the serum UA in the model group was significantly increased (^###^ *p* < 0.001), indicating that the model construction method used was effective and successful. The serum-UA levels of mice in different treatment groups showed a decreasing trend after gavage administration, among which the positive control group and the HPO group showed excellent UA-lowering efficacy (*** *p* < 0.001); no statistically significant difference was observed in the comparison between the groups (Figure 5A).

Since the level of XOD activity is proportional to the rate of UA production, we measured the XOD activity in the serums of mice to reflect the level of XOD inhibition of the drug. The results of the XOD activity assay showed that the treatment of yeast-paste gavage modeling resulted in a significant effect on the XOD activity of the mice in each group (^###^ *p* < 0.001); this is because, after the mice received yeast paste gastric gavage, the purine-like substances in the body accumulated in large quantities, which led to the elevation of XOD activity in the body and the production of large quantities of UA. The XOD-inhibitor allopurinol group and the HPO group showed significantly inhibited levels of XOD activity and lowered serum-UA levels, and there were no statistically significant differences compared with the control group (Figure 5B).

### 3.7. Renal Protection Properties of PO

**Figure 6:**
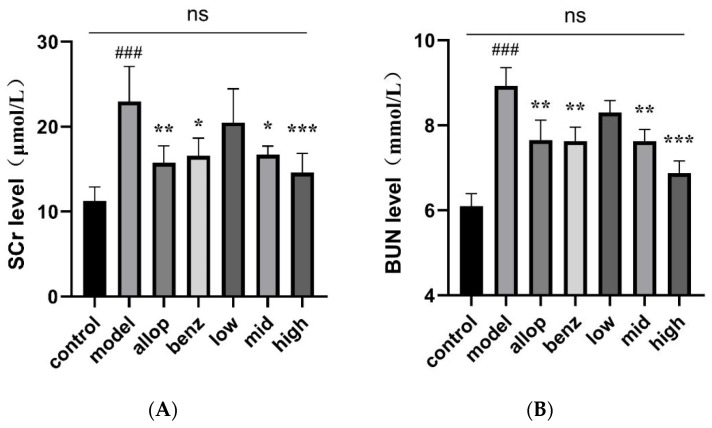
Serum levels of SCr and BUN in mice after intragastric administration. (**A**) The level of SCr in serum of mice after intragastric administration. (**B**) The level of BUN in serum of mice after intragastric administration. ^###^ *p* < 0.001 compared with the control group. * *p* < 0.05, ** *p* < 0.01, and *** *p* < 0.001, compared with the model group. ns (no significance).

The centrifuged serums were immediately, and following the operating instructions in the biochemical kit, tested for the determination of SCr levels, as shown in Figure 6A; the SCr values in the model group of mice showed a significantly higher trend compared with the normal group (^###^ *p* < 0.001), and after the administration of the drug, the SCr levels in the allopurinol group, the benzbromarone group, the MPO group, and the HPO group were significantly decreased (* *p* < 0.05, ** *p* < 0.01, *** *p* < 0.001), and there was no significant difference in SCr value between the HPO group and the control group. BUN levels were measured by the 96-well plate colorimetric method (Figure 6B), and from the results returned by the biochemical assay kit, it could be seen that the BUN levels of mice in the model group were significantly higher than those in the control group (^###^ *p* < 0.001), while after the administration of the drug, the BUN levels of the mice in all the groups appeared to be reduced to varying degrees; the dose-dependencies between the various administration groups of PO are presented, and the BUN levels of the HPO group can be seen to be significantly lower than those of the model group (*** *p* < 0.001).

### 3.8. Effects of PO on Renal Histopathology

**Figure 7:**
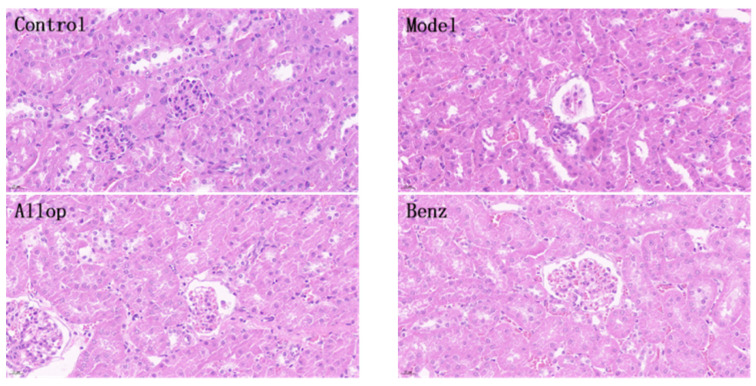

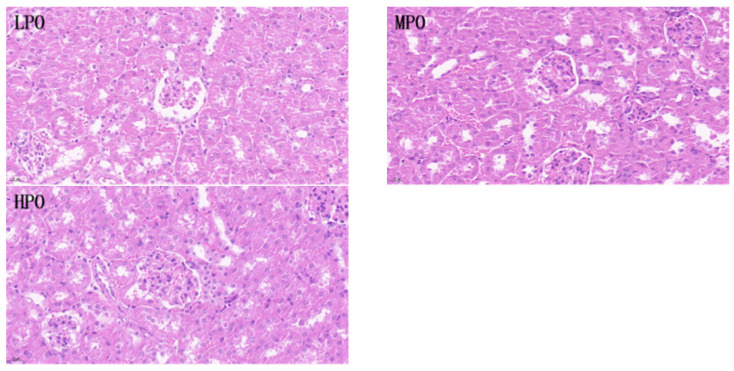
H/E-stained pathological sections of mouse kidney tissue (400×).

Illustrated in Figure 7, the histological analysis of renal tissues from individual mouse cohorts subsequent to H/E staining illuminated that within the control cohort, renal tubules manifested an exemplary preservation of morphology, which was characterized by proximal epithelial cells presenting a plump appearance and the renal interstitia being densely populated. Nonetheless, subsequent to gastric gavage with yeast paste to instigate the experimental model, the experimental cohort displayed conspicuous eosinophilia alongside pronounced tubular vacuolization. Analogous eosinophilic modifications, coupled with a degree of tubular vacuolization, were discernible within the allopurinol cohort, the benzbromarone cohort, and the LPO cohort, coinciding with a concurrent relaxation of tissue architecture. Conversely, there was an absence of noteworthy morphological aberrations concerning tubular cell integrity and tissue structure within the MPO and HPO cohorts when juxtaposed with the pristine control cohort.

### 3.9. Western Blot Analysis of Kidney Tissues

**Figure 8:**
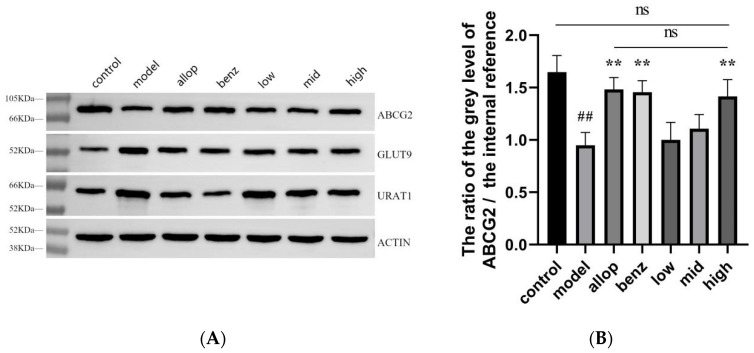

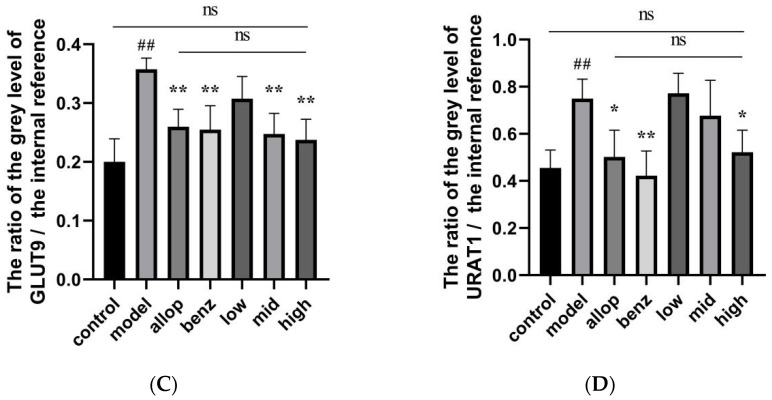
(**A**) Western Blot analysis of kidney tissues after drug administration in each group of mice. (**B**) ABCG2 protein expression level. (**C**) GLUT9 protein expression level. (**D**) URAT1 protein expression level. ^##^ *p* < 0.01 compared with the control group. * *p* < 0.05 and ** *p* < 0.01, compared with the model. ns (no significance).

The expression level of urate transporters in the kidney tissue for each group of mice was obtained by Western Blot, as shown in Figure 8A. The relative content of the protein sample can be obtained by comparing the gray value of the protein index with the gray value of the internal reference band. By analyzing these data, the expression level of urate transporter in the kidney tissue of HUA mice was explored. Compared with the normal group, the expression of ABCG2 protein in the model group was significantly decreased (^##^ *p* < 0.01), while the expression intensity of ABCG2 protein in the PO group was dose-dependent, and there was no significant difference in the expression level between the high-dose group and the normal group, indicating that PO can increase the excretion of UA in mice by promoting the expression of ABCG2 protein (Figure 8B). Compared with the model group, GLUT9 protein levels were significantly down-regulated in the allopurinol group, benzbromarone group, MPO group, and HPO group (** *p* < 0.01) (Figure 8C). Compared with the control group, the expression of URAT1 protein in the model group was significantly up-regulated (^##^ *p* < 0.01), while benzbromarone, as an inhibitor of URAT1 protein, significantly down-regulated its expression level. In addition, the PO administration group showed a dose-dependent downward trend, and there was no statistical difference in the expression level of URAT1 protein between the HPO group and the control group (Figure 8D).

### 3.10. Effects of PO on Urate Transport Proteins

IHC analysis was performed on the kidney tissue sections of mice in each experimental group. The brown specific staining area observed by the microscope indicated the positive reaction of antigen–antibody binding, while the light yellow area represented the background color. According to the staining area and depth, the intensity of antigen–antibody binding can be intuitively reflected.

**Figure 9:**
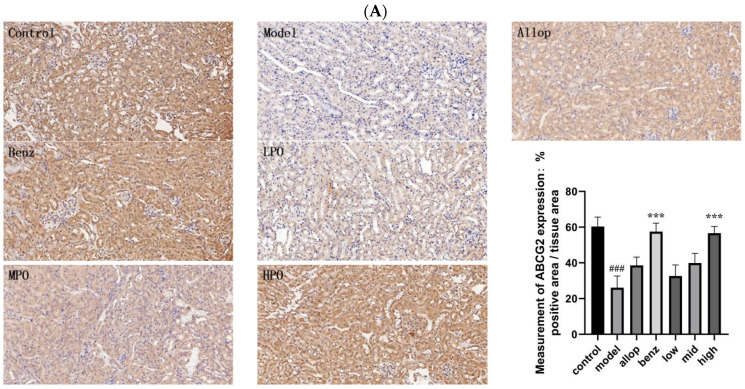

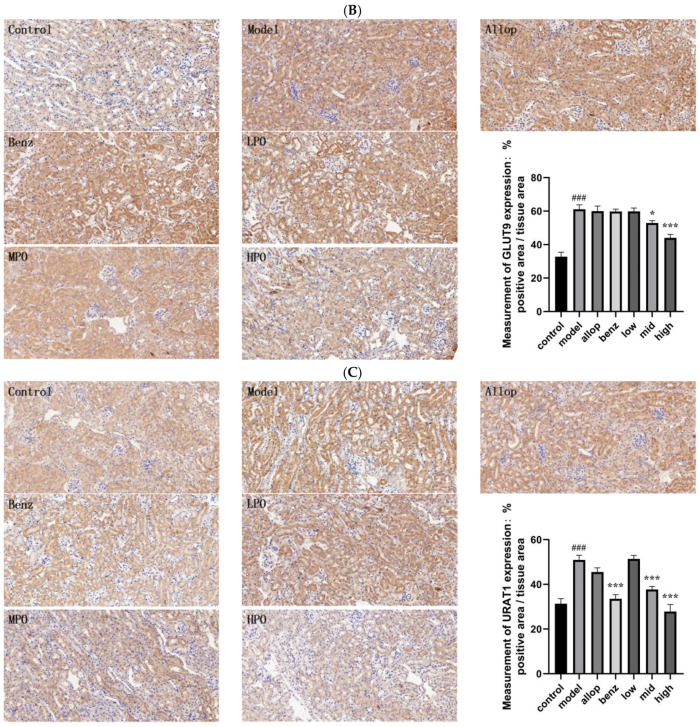
IHC analysis of renal tissues after drug administration in each group of mice (200×). (**A**) ABCG2 protein IHC expression level. (**B**) GLUT9 protein IHC expression level. (**C**) URAT1 protein IHC expression level. ^###^ *p* < 0.001 vs. control. * *p* < 0.05 and *** *p* < 0.001, vs. model. Positive area values were analyzed by Image.

As shown in Figure 9A–C, the renal cortex region showed different degrees of positive reaction. URAT1 protein was widely expressed in renal tubular cells, and the expression levels of the benzbromarone group and HPO group were significantly lower than that of the model group. The ABCG2 protein was widely expressed in the cytoplasm of the renal tubular epithelial cells in each group. Compared with the model group, the control group and the HPO group had stronger positive localization.

IHC results showed that the ABCG2 protein was widely distributed in the kidney of PO-treated mice, covering the renal cortex and renal tubular cells, and was more dense than that of the HUA model mice in the control group. This indicates that the ABCG2 protein can maintain the homeostasis of UA in the body by promoting UA excretion. In the IHC test results of the HPO group, the expression intensity levels of the URAT1 and GLUT9 proteins were the same as those shown in the Western Blot results, further confirming that the HPO group indeed exerted a significant regulatory effect on the urate transporters.

## 4. Discussion

Currently, allopurinol, benzbromarone, febuxostat, and other drugs are extensively utilized in the clinical management of HUA [19,57]. Nevertheless, due to varying degrees of adverse reactions associated with these agents, there is a growing interest in natural remedies with diminished side effects [58,59]. Studies have found that many plants that people eat daily have been found to have uric acid-lowering effects [60]. In addition, some traditional Chinese herbal medicines have been shown to have some effect in alleviating hyperuricemia, especially in the protection of liver and kidney function [61]. Among them, many plant-based active ingredients such as flavonoids, phenolic acids, saponins, etc., have been shown to reduce uric acid levels in a variety of ways without obvious side effects [62,63]. In summary, plant-derived functional foods and their active ingredients have shown potential in the treatment of hyperuricemia. These findings provide a treatment plan with small side effects, good therapeutic effects, and easier acceptance for people, and one which may become a safe and effective natural alternative therapy for hyperuricemia.

Studies have shown that the same plant in different habitats may have different pharmacological effects, and the theory of dao-di herbs is also mentioned in the field of traditional Chinese medicine [64,65]. In order to further study the active substances and pharmacological effects of PO, we meticulously procured fresh PO samples from four provinces in China with great climate differences, including Sichuan, Henan, Guangdong, and Jilin, for subsequent extraction and purification. Employing LC-MS technology alongside the XOD enzymatic reaction system enabled thorough identification and comparative analysis. Notably, findings revealed a marginally superior XOD inhibition efficacy in Sichuan specimens compared to samples of the plant collected from other habitats. Studies have reported that different conditions of cultivation and crop management lead to changes in PO’s active ingredients [27,66]. At the same time, our findings also reveal that different geographical and climatic conditions may be one of the reasons for the changes in bioactive substances in PO, which also confirms the dao-di herbs theory in the field of traditional Chinese medicine.

Furthermore, subsequent to the analysis of Liquid Chromatography-Mass Spectrometry (LC-MS) identification outcomes and the review of pertinent literature, berberine and stachydrine were discerned within Sichuan-derived PO. Subsequently, we verified the presence of these two components in PO by standard control. This is the first time that berberine and stachydrine were identified from PO. Many studies have shown that berberine [55,67,68,69] and stachydrine [56,70,71,72] have a variety of physiological activities, which lays a theoretical foundation for further study of the effective components of PO and the related mechanism of network pharmacology. At the same time, the results of molecular docking showed that berberine had strong docking activity with XOD, and the studies reported in [73,74] also showed that berberine had the effect of reducing uric acid. Therefore, we speculated that berberine was one of the active substances of PO in the reduction of uric acid.

With the development of modern society, people‘s eating habits have gradually shown a trend of high levels of purine [75]. Studies have shown that the risk of hyperuricemia is positively correlated with red meat, seafood, alcohol, and fructose intake [76]. In order to more accurately replicate the physiological conversion of exogenous purines into UA within the human body, we employed the technique of gavaging yeast paste to establish an experimental model of HUA mice [54]. In addition, we selected the XOD inhibitor allopurinol and the uric acid-promoting drug benzbromarone as two positive drug groups to better study the uric acid-lowering mechanism of PO. By assessing serum biochemical indices in the murine bloodstream, we observed a gradual normalization of serum-UA levels in mice administered medium (1 g/kg) and high (2 g/kg) doses of PO after one week (** *p* < 0.01), with no statistical variance compared to either the allopurinol or benzbromarone groups. Furthermore, compared with the model group, the content levels of SCr and BUN also decreased significantly (** *p* < 0.01), and the BUN level in the HPO group was more similar to that in the control group, which indicated that PO had weaker hepatorenal toxicity than did allopurinol or benzbromarone. Studies have shown that elevated serum-UA levels are a risk factor for decreased renal function, and elevated UA levels can lead to a series of immune and inflammatory responses [77,78]. Our in vivo results showed that PO extract effectively inhibited XOD activity, and potential renal tissue damage caused by elevated UA levels was effectively alleviated in mice.

At present, XOD inhibitors and uric acid excretory drugs are often used in clinical treatment of gout patients, but these drugs can also cause certain forms of damage to the kidney [79,80]. Allopurinol can inhibit XOD activity and prevent the conversion of hypoxanthine and xanthine to UA [81]. Benzbromarone can promote UA excretion by inhibiting URAT1 transporters [82]. In vivo experiments and serum XOD activity analysis showed that the XOD activity of the HPO group (** *p* < 0.01) was significantly reduced, and there was no significant difference from the allopurinol group, indicating that the HPO group had good XOD inhibition performance. Through histological examination utilizing H/E staining of murine renal tissues, the model group exhibited notable pathological alterations, including eosinophilic degeneration resultant from urate accumulation and pronounced dilatation of renal tubules. Conversely, the MPO and HPO groups displayed no discernible morphological aberrations within renal tubular cells or tissue architecture, which indicated that the PO extract had no obvious nephrotoxicity. The above results show that PO extract is safer than allopurinol and benzbromarone and has better clinical application value.

Studies have shown that various urate transporters in the kidney are involved in the regulation of serum-UA levels [83]. Investigation into hyperuricemia-induced renal tissue in mice revealed that PO exerts efficacious synergistic regulation over UA excretion protein ABCG2 and urate transporters URAT1 and GLUT9, thereby preserving murine UA homeostasis. Consequently, we posit that PO has the dual capacity to attenuate UA synthesis by suppressing XOD activity and to diminish UA reabsorption while facilitating its excretion by modulating the overexpression of urate transporters and bolstering UA excretion proteins. In general, PO effectively reduces serum-UA levels through the synergistic effects of various components, targets, and pathways, thereby maintaining UA homeostasis.

## 5. Conclusions

In this study, we first screened the PO from Sichuan, determining that it demonstrated the best in vitro XOD inhibitory activity among the plant samples from the four habitats, and berberine and stachydrine were isolated and identified from this PO for the first time. This finding reveals that different geographical and climatic conditions may be one of the reasons for the changes of bioactive substances in PO. Then ABCG2, URAT1, and other proteins were screened out as research targets by network pharmacology. Molecular docking prediction and in vitro verification showed that berberine had strong docking activity with XOD. Finally, in vivo results showed that PO could inhibit excessive uric acid production and promote uric acid excretion by inhibiting XOD activity, inhibiting the expression of uric acid transporters (GLUT9, URAT1) and promoting the expression of uric acid excretion protein (ABCG2), and thereby effectively reducing serum-UA levels in mouse models. In addition, compared with positive drugs, PO extracts showed less nephrotoxicity and increased safety. In summary, our study showed the potential of purslane to reduce uric acid, and provided a theoretical basis for the development of the edible wild plant purslane.

## 6. Patents

An extraction method of effective components of purslane and its application in reducing uric acid. Bo Gao; Yiming Zhang; Fei Ye; Ming Kang; Zhenlong Ge; Shengying Zhu; Yanjing Feng; Hao Chang. ZL 2024 1 0543667.X.

## Data Availability

Data are contained within the article and Supplementary Materials.

## Supplementary Materials

The following supporting information can be downloaded at: https://www.mdpi.com/article/10.3390/nu16203549/s1, Figure S1: Berberine in vitro XOD inhibition-ability experiment.
