# Supplementary figures and images for: Network Pharmacology Combined with Experimental Validation to Investigate the Mechanism of the Anti-Hyperuricemia Action of Portulaca oleracea Extract
Source: Nutrients. 2024 Oct 19;16(20):3549. doi: 10.3390/nu16203549 (PMC11510147; doi:10.3390/nu16203549)

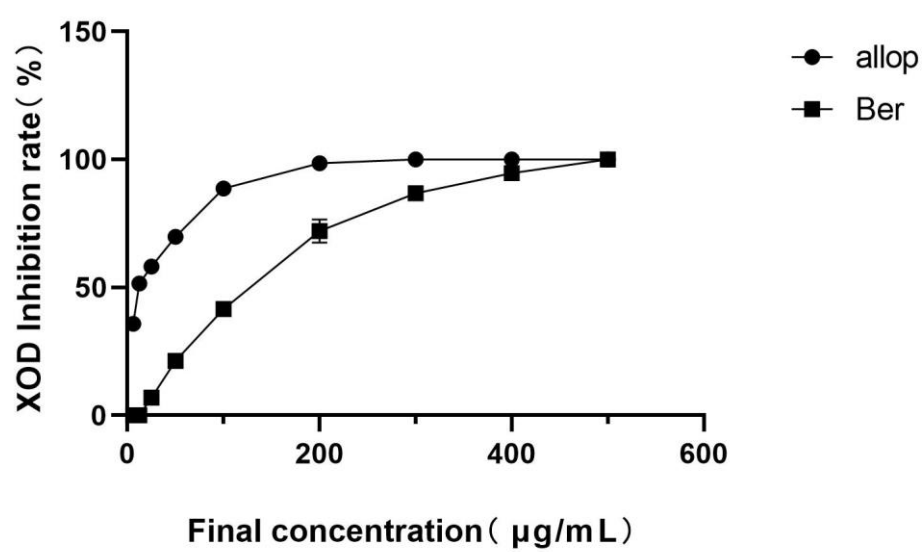

**Fig. S1** The inhibitory effect of berberine on XOD in vitro.

Supplement: Supplementary file 1 [file nutrients-16-03549-s001.zip › nutrients-3225355-supplementary.pdf]
